# Real-World Effectiveness and Safety of Upadacitinib in Patients with Ulcerative Colitis: A Systematic Review and Meta-Analysis

**DOI:** 10.3390/jcm14072232

**Published:** 2025-03-25

**Authors:** Carlos Taxonera, Miguel A. García-Brenes, María Machín, David Olivares, Olga N. López-García, Raúl Zapater, Cristina Alba

**Affiliations:** 1 Inflammatory Bowel Disease Unit, Gastroenterology Department, Hospital Clínico San Carlos, 28040 Madrid, Spain; mgbrenes.externo@salud.madrid.org (M.A.G.-B.); mmachin@salud.madrid.org (M.M.); oliapunto@gmail.com (O.N.L.-G.); raul.zapater@salud.madrid.org (R.Z.); cristina.alba@telefonica.net (C.A.); 2 Instituto de Investigación del Hospital Clínico San Carlos [IdISSC], 28040 Madrid, Spain; 3 National Center of Epidemiology, Instituto de Salud Carlos III, 28029 Madrid, Spain; dolivares@isciii.es

**Keywords:** upadacitinib, Janus kinase, ulcerative colitis, meta-analysis, real-world effectiveness, safety

## Abstract

**Background/Objectives**: Evidence is needed on the real-world outcomes of upadacitinib in patients with ulcerative colitis. This systematic review and meta-analysis evaluated the real-world effectiveness of upadacitinib for active UC. **Methods**: The primary outcome was clinical remission evaluated at week 8. Secondary outcomes included response, steroid-free remission, biochemical remission, colectomy, and safety. A random-effects meta-analysis model was used to calculate the pooled effect sizes (percentages or incidence rates) of effectiveness and safety outcomes. **Results**: Twenty-four studies with 1388 patients were included. Ninety-four percent of patients had previously failed biologics or Janus kinase inhibitors (JAKi), including 53.2% with tofacitinib. Clinical remission at week 8 was achieved in 68.4% of patients (95% confidence interval 55.5–80.2). Clinical remission was achieved in 48.3%, 71.1%, and 64.6% of patients at weeks 2 to 6, 12 to 16, and 24 to 36, respectively. Response was achieved in 72.6%, 82.1%, and 78.7% of patients at weeks 2 to 6, week 8, and weeks 12 to 16, respectively. Steroid-free remission was achieved in 39% of patients at week 8. Upadacitinib results were unaffected by prior biologic or JAKi failure. Mean fecal calprotectin level decreased from 1485.0 µ/g at baseline to 454.8 µ/g post-treatment (*p* < 0.01). The mean CRP level decreased from 12.3 mg/L at baseline to 4.4 mg/L post-treatment (*p* = 0.02). The incidence rates of colectomy, serious adverse events, and herpes zoster were 13.3, 2.3, and 1.7 per 100 patient-years, respectively. **Conclusions**: This meta-analysis confirms the effectiveness and safety of upadacitinib in a highly treatment-refractory population of UC patients.

## 1. Introduction

Upadacitinib is an oral second-generation selective Janus kinase inhibitor that operates by inhibiting intracellular cytoplasmic enzymes known as Janus kinase (JAK), a family of four tyrosine kinases (JAK1, JAK2, JAK3, and TIK2), modulating inflammatory pathways. Upadacitinib has a selective and more prominent inhibitory effect on JAK1 compared with the other subtypes [1]. Several cytokines bind to their receptors and activate intracellular JAKs in pairs, leading to the phosphorylation of Signal Transducers and Activators of Transcription (STATs) [2]. The phosphorylated and dimerized STATs subsequently move to the cell nucleus, where they influence gene transcription. This process affects cellular functions and regulates signaling for critical inflammatory mediators such as interferon-γ, type I interferon, and various interleukins. Unlike biologics, JAK inhibitors modulate multiple cytokine pathways by targeting common signaling mechanisms [3].

JAK inhibition can lead to adverse effects such as cytopenia and infectious complications due to a blockade of GM-CSF and IFN-g signaling, respectively. As JAK2 activity is critical for hematopoiesis, its inhibition could lead to reduced erythrocyte and leukocyte counts. In addition, JAK2 inhibition can cause thrombocytosis and has been associated with deep vein thrombosis and pulmonary embolism. The aim of inhibitors is not to block the JAK pathway completely, but to reversibly reduce the activity of one or more JAK isoforms, so that the effects can be reversed when the drug is discontinued. Selective JAK1 inhibitors have been developed to minimize potential adverse effects [3,4]. However, because JAKs often bind in pairs of two different isoforms (except for JAK2, which can bind to itself), the selectivity of a JAK inhibitor is considered to be relative. Therefore, it may be more accurate to refer to them as “preferential” rather than “selective” JAK inhibitors.

The phase 3 randomized controlled trials (RCTs) evaluating induction of upadacitinib 45 mg once daily (U-ACHIEVE induction [UC1] and U-ACCOMPLISH [UC2]), and maintenance of upadacitinib 30 or upadacitinib 15 mg once daily (U-ACHIEVE maintenance [UC3]) demonstrated that in patients with moderate-to-severely active UC, upadacitinib was effective for induction of remission at 8 weeks (inductions studies) and 52 weeks (maintenance study), with key secondary endpoints of endoscopic remission, clinical response, and histological–endoscopic mucosal improvement [4]. While there were concerns about potential side effects of JAKi due to the diverse functions of JAK-STAT signaling pathways, the safety profile of upadacitinib in UC was deemed acceptable based on an ongoing Phase 3, open-label, 288-week long-term extension (LTE) study [5].

Strict inclusion criteria in randomized controlled trials (RCTs) can limit the patient population and reduce the applicability of results to clinical practice. This issue is particularly pertinent in populations with inflammatory bowel disease. Therefore, observational studies assessing effectiveness and safety are essential to validate the clinical benefits observed in trials and to inform therapeutic decisions [6]. Currently, gastroenterologists have access to several advanced therapies for moderate-to-severe UC, including three tumor necrosis factor (TNF) antibodies, ustekinumab (IL 12–23 antibody), mirikizumab (IL 23 antibody), the anti-integrin vedolizumab, the sphingosine-1-phosphate receptor modulators ozanimod and etrasimod, and the JAK inhibitors tofacitinib, filgotinib, and upadacitinib. Considering the wide range of available therapeutic options, evaluating the real-world effectiveness and safety of upadacitinib is crucial to inform therapeutic decision making. Therefore, several observational studies have been conducted in recent years to evaluate the outcomes of upadacitinib in UC [7,8,9]. A meta-analysis, particularly with large patient populations, can enhance the reliability of these observations.

The aim of this systematic review of observational studies was to summarize reported evidence on the real-world outcomes of upadacitinib for UC and to conduct a meta-analysis of effectiveness and safety data.

## 2. Materials and Methods

This meta-analysis adheres to the guidelines of the updated PRISMA 2020 statement (flowchart) and the MOOSE Checklist for Meta-analyses of Observational Studies [10]. The protocol is registered in PROSPERO (CRD42024555177).

### 2.1. Search Strategy and Study Selection

A systematic search was conducted in PubMed, Web of Science, EMBASE, ScienceDirect, and Scopus up to 1 November 2024, to identify relevant studies reporting outcomes of upadacitinib for UC. Both prospective and retrospective observational studies, including full-text publications and abstracts, were included. Studies with fewer than five participants and all randomized and non-randomized controlled clinical trials were excluded from the analysis. To ensure a comprehensive and inclusive search strategy, we used the following key terms across the databases: “ulcerative colitis” (as medical subject heading and as free-text term) combined using the Boolean operator ‘AND’ with studies identified with the terms “upadacitinib”, “janus kinase inhibitor”, “JAK inhibitor”, “JAK-STAT” and “Rinvoq” with no language or date restrictions (Supplementary Table S1). Additionally, backward citation tracking of references was conducted manually to find any more studies. Two investigators, M.A.G.-B. and M.M., independently screened citations, resolving discrepancies with a third investigator (C.T.).

### 2.2. Data Extraction and Outcome Measures

Data were extracted into a Microsoft Excel spreadsheet (XP professional edition; Microsoft Corp, Redmond, WA, USA) by two investigators (M.A.G.-B. and M.M.), with a third investigator (C.T.) checking for accuracy. We collected data from each eligible study including author’s name, publication year, country, type of publication, study design, number of patients, demographics, UC characteristics, prior advanced treatments, current steroids, and clinical outcomes. The primary outcome was clinical remission at week 8. Secondary outcomes included clinical remission at other timepoints, clinical response, steroid-free clinical remission (SFCR), biochemical remission (fecal calprotectin and C-reactive protein [CRP] levels), safety, and colectomy rate. Outcomes were evaluated at weeks 2 to 6, 8, 12 to 16, and 24 to 36, where available. We performed subgroup analysis for clinical remission rate at week 8 to evaluate effect modifications by covariates. Assessment of clinical outcomes, including calprotectin or CRP biochemical remission rates, were based on the definitions provided by each study. Colectomies were reported separately, not as adverse events.

### 2.3. Quality Assessment

The Joanna Briggs Institute (JBI) Critical Appraisal tool, consisting of 10 domains, was used to assess study quality and bias. Responses included “yes”, “no”, “unclear”, and “not applicable”. Studies were classified by risk of bias: low (≥70% “yes” answers), moderate (50–69%), and high (<50%) [11]. Two independent investigators (M.A.G.-B. and M.M.) conducted the assessments, with a third investigator (C.T.) resolving disagreements.

### 2.4. Statistical Analysis

Data from each study were pooled to calculate the effect sizes for outcomes using the DerSimonian and Laird inverse variance weighting method to account for between-study heterogeneity [12]. A random-effects meta-analysis model was used to calculate the pooled rates of clinical and safety outcomes. Outcomes were estimated as pooled percentages or incidence rates (IRs) with 95% confidence intervals (CI) and depicted in forest plots. The I^2^ statistics were used to assess study heterogeneity as per the Cochrane Handbook guidelines: 0–40% indicates low importance, 30–60% moderate heterogeneity, 50–90% substantial heterogeneity, and 75–100% considerable heterogeneity [13]. The variability in effect estimates due to heterogeneity was evaluated with the Cochrane Q test, with a *p*-value < 0.10 considered statistically significant. For outcomes reported in nine or more studies, publication bias was assessed using Egger weighted regression, with *p*-value < 0.05 indicating bias. To guarantee the reliability of our findings, a sensitivity analysis was performed to assess the impact of each individual study on the cumulative effect size for the primary endpoint. The need for colectomy and adverse events were reported both as the total number of specified events overall and as incidence rates (IRs) per 100 patients-year (PY) of exposure. Follow-up times provided by authors as median and interquartile ranges were converted into means and standard deviations [14]. The mean follow-up time and the mean fecal calprotectin and CRP levels were calculated as means of means. We considered a *p*-value of 0.05 or lower as statistically significant unless otherwise stated. The meta-analysis was performed using the metaprop and metarate command of the metapackage in R (version 4.3.3) [15].

## 3. Results

**Figure 1:**
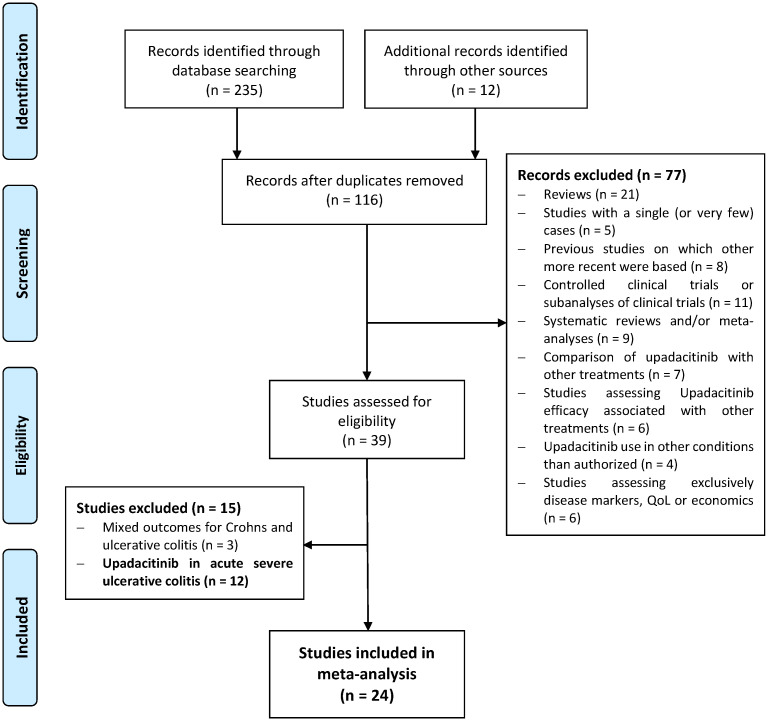
PRISMA flow diagram of the study selection.

The flowchart of the study selection is shown in Figure 1. Of the 247 citations identified, 24 studies including 1388 patients with UC were eligible for inclusion in this systematic review [7,8,9,16,17,18,19,20,21,22,23,24,25,26,27,28,29,30,31,32,33,34,35,36]. The characteristics of the studies and the outcomes evaluated in each study are shown in Table S2. Four studies were presented as full-text articles, and twenty were conference proceedings (abstracts or reports). According to JBI quality assessment criteria, seventeen studies had a low risk of bias, and seven articles had a moderate risk (Table S3). In two studies, including 45 patients, only demographic data were used [21,25]. Therefore, 22 studies were included in the meta-analysis for efficacy outcomes. Definitions of clinical remission, SFCR, clinical response and biochemical remission are summarized in Table S4.

### 3.1. Demographics and Characteristics of UC Populations

Demographics of populations, characteristics of UC, and previous advanced treatments are presented in Table 1. The mean follow-up time was 19 weeks. Among the patients, 69.5% had extensive UC, with a mean disease duration of 8.3 years. The patient population was highly refractory to treatments, with 94.6% having previously failed biologic or JAKi therapies. These included anti-TNF (78.7%), vedolizumab (53.6%), ustekinumab (35.1%), and tofacitinib (53.2%). During the induction phase, 94.6% of patients received upadacitinib 45 mg once daily, and 44.5% received concomitant steroids. After induction, the maintenance dose of upadacitinib was adjusted to 45 mg (25.8%), 30 mg (72.4%), or 15 mg (6.0%) according to patient needs.

### 3.2. Primary Endpoint: Clinical Remission

**Figure 2:**
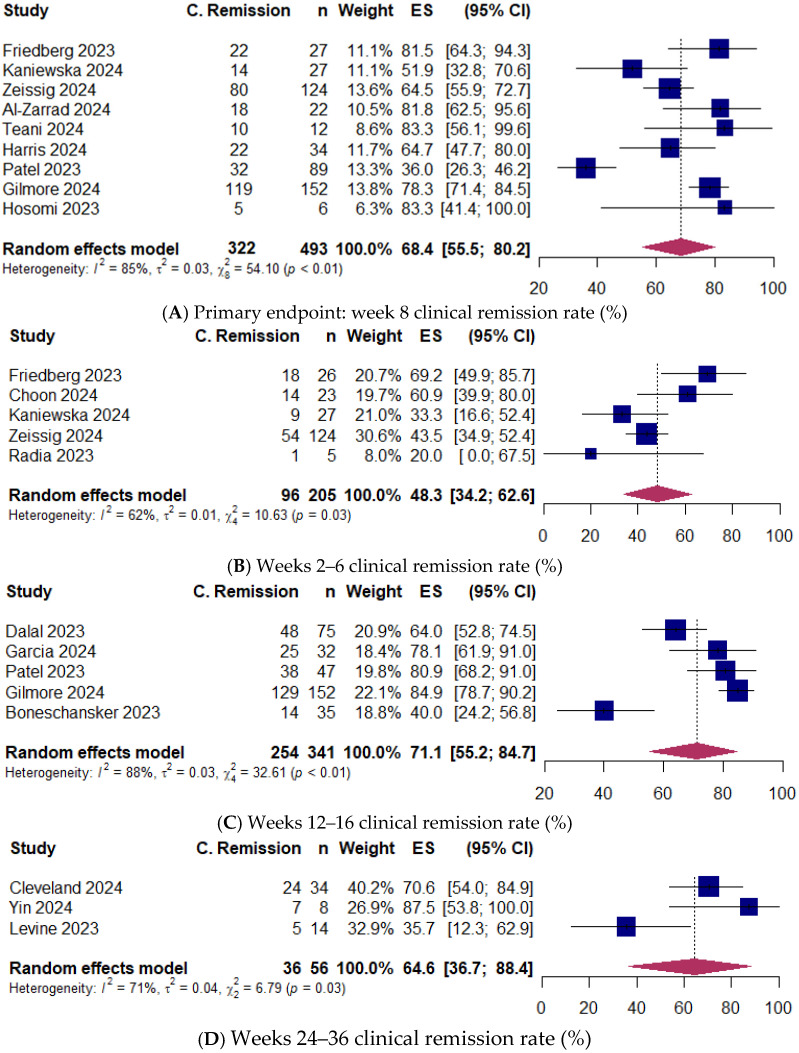
(**A**) Primary endpoint: clinical remission rate at week 8. Egger weighted regression, *p* = 0.33 [8,18,19,20,22,24,26,30,33]. (**B**) Clinical remission rate at weeks 2–6 [8,17,18,19,34]. (**C**) Clinical remission rate at weeks 12–16 [7,23,26,30,31]. (**D**) Clinical remission rate at weeks 24–36 [9,16,32]. Random-effects model was applied. ES, effect size; CI, confidence interval.

Eighteen studies [7,8,9,16,17,18,19,20,22,23,24,26,30,31,32,33,34,35] (746 patients) reported clinical remission rates (Table S2). The pooled proportion of patients achieving clinical remission at week 8 (primary endpoint) was 68.4% (95% CI 55.5–80.2) [493 patients; 9 studies: [8,18,19,20,22,24,26,30,33,35]] (Figure 2). At weeks 2 to 6, 48.3% of patients (95% CI 34.2–62.6) [205 patients; 5 studies: [8,17,18,19,34]] achieved clinical remission. Clinical remission was observed in 71.1% of patients (95% CI 55.2–84.7) [341 patients; 5 studies: [7,23,26,30,31]] at weeks 12 to 16, and in 64.6% of patients (95% CI 36.7–88.4) [56 patients; 3 studies: [9,16,32]] at weeks 24 to 36. Substantial between-study heterogeneity was observed at week 8 (I^2^ = 85%), weeks 2 to 6 (I^2^ = 62%), weeks 12 to 16 (I^2^ = 88%), and weeks 24 to 36 (I^2^ = 71%) (Figure 2). The Egger regression test indicated no publication bias at week 8 (*p* = 0.33).

### 3.3. Clinical Response

Twelve studies [7,8,17,20,22,24,26,27,28,31,34,36] including 496 patients reported clinical response rates (Table S2). Response was achieved in 72.6% of patients at weeks 2 to 6 (95% CI 58.8–84.8) [53 patients; 3 studies: [8,17,34]], and in 82.1% at week 8 (95% CI 76.4–87.3) [210 patients; 6 studies: [8.20,22,24,26,36]] (Supplementary Figure S1). At weeks 12 to 16, 78.7% of patients (95% CI 66.3–89.1) [257 patients; 5 studies: [7,26,27,28,31]] had a clinical response. Heterogeneity between studies might not be important at weeks 2 to 6 and week 8 (both I^2^ = 0%), and substantial at weeks 12 to 16 (I^2^ = 77%) (Figure S1).

### 3.4. Steroid-Free Clinical Remission

Three studies [8,19,36] with 177 patients reported that 39.4% achieved SFCR at week 8 (95% CI 17.2–64.0) (Figure S2). Significant between-study heterogeneity was observed (I^2^ = 87%).

### 3.5. Biochemical Remission

**Figure 3:**
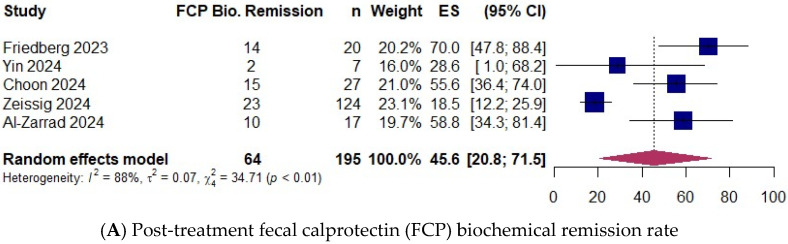

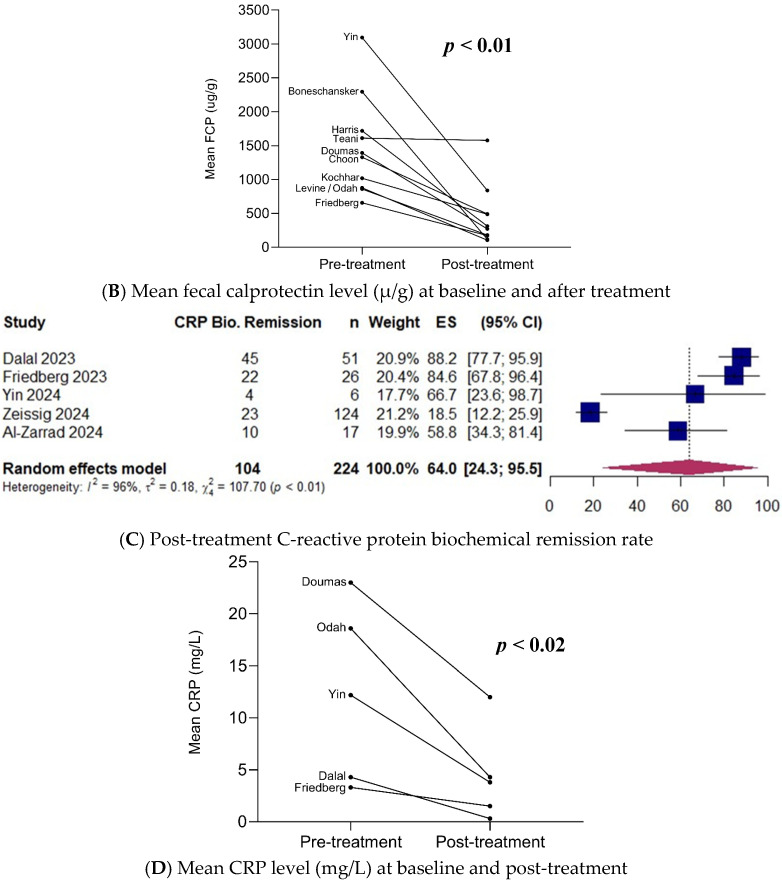
(**A**) Post-treatment fecal calprotectin (FCP) biochemical remission rate (random-effects model) [8,16,17,19,20]. (**B**) Mean fecal calprotectin level (µ/g) at baseline and post-treatment. (**C**) Post-treatment C-reactive protein (CRP) biochemical remission rate (random-effects model) [7,8,16,19,20]. (**D**) Mean CRP level (mg/L) at baseline and post-treatment. ES, effect size; CI, confidence interval.

Six studies [7,8,16,17,19,20] with 313 patients reported the biochemical remission rate of fecal calprotectin or CRP as defined by each study (Table S4). Fecal calprotectin and CRP samples were generally collected at week 8 of treatment, defined as post-treatment. In the studies by Zeissig et al. [19] and Al-Zarrad et al. [20], biochemical remission was defined as the combined biochemical remission rate of both calprotectin and CRP. The pooled proportion of patients who achieved post-treatment fecal calprotectin biochemical remission was 45.6% (95% CI 20.8–71.5) [195 patients; 5 studies: [8,16,17,19,20]] (Figure 3A). The proportion of patients achieving CRP biochemical remission following treatment was 64.0% (95% CI 24.3–95.5) [224 patients; 5 studies: [7,8,16,19,20]] (Figure 3C). The mean fecal calprotectin level decreased from 1485.0 µ/g at baseline to 454.8 µ/g post-treatment (*p* < 0.01) [261 patients; 10 studies: [8,16,17,22,24,28,29,31,32,36]] (Figure 3B). Additionally, the mean CRP level decreased from 12.3 mg/L at baseline to 4.4 mg/L post-treatment (*p* = 0.02) [169 patients; 5 studies: [7,8,16,28,36]] (Figure 3D).

### 3.6. Clinical Remission with Upadacitinib as Second-Line JAKi

Five studies [7,30,31,33,35] reported clinical remission rates in cohorts that failed a first JAKi, mainly tofacitinib (Table S2). Reasons for failure of the first JAKi included primary non-response (39.6%), secondary loss of response (35.4%), partial response (17.5%), and adverse events (7.6%). Clinical remission with upadacitinib as a second-line JAKi was achieved in 76.3% of patients at week 8 (95% CI 63.1–87.6) [56 patients; 3 studies: [30,33,35]], and in 69.3% at weeks 12 to16 (95% CI 36.7–95.2) [77 patients; 4 studies: [7,30,31,35]] (Figure S3). Between-study heterogeneity might not be important at week 8 (I^2^ = 0%), and substantial at weeks 12 to 16 (I^2^ = 75%) (Figure S3).

### 3.7. Subgroup Analysis

Analysis for clinical remission rate at week 8 was performed for the following subgroups: study design (prospective vs. retrospective), study site (single center vs. multi-center), percentage of patients with prior exposure to biologics (100% vs. ≤85%), and studies with 100% prior exposure to JAKi (No vs. Yes) (Figure S4).

Subgroup analysis identified a statistically significant effect based on the percentage of patients with prior biologic exposure (*p* = 0.03). (Figure S4C). At week 8, clinical remission rate was 83.0% in the 100% bio-exposed group (95% CI 69.5–93.7) [45 patients; 3 studies: [8,22,33]], compared to 54.8% in the subgroup with ≤85% exposure to biologics (95% CI 34.4–74.3) [247 patients; 3 studies: [19,24,26]].

The analysis further indicates that there are no statistically significant effects in any of the other subgroups studied, indicating that none of these covariates modify the treatment effect (Figure S4A,B,D).

### 3.8. Colectomy Rates

**Figure 4:**
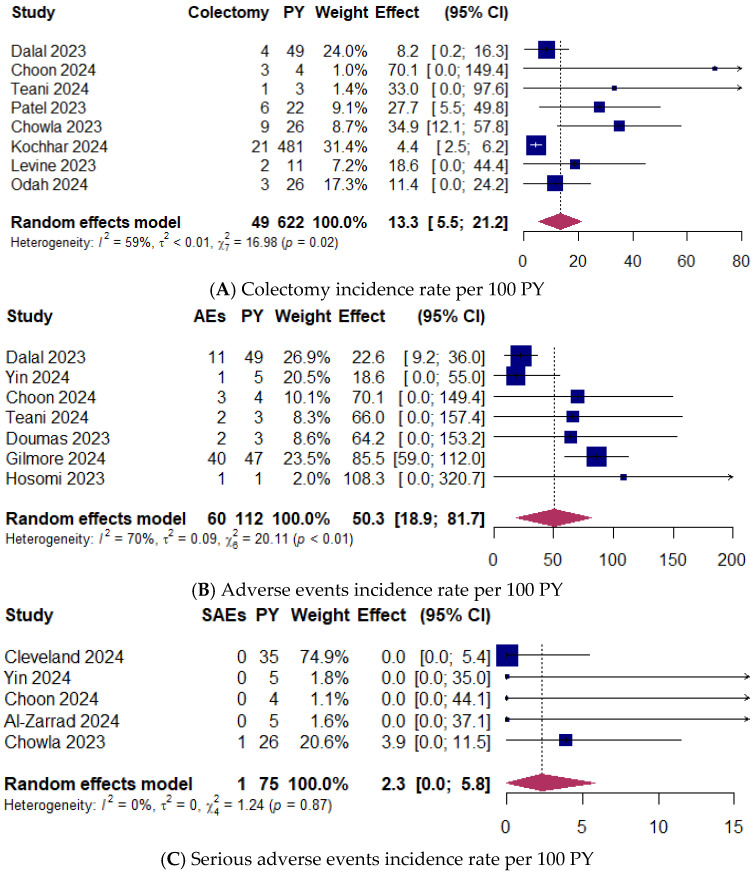

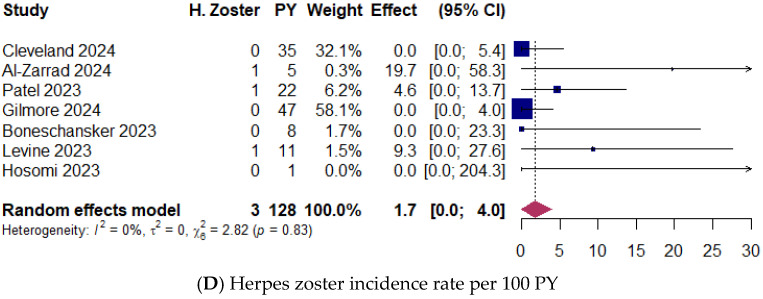
(**A**) Colectomy incidence rate per 100 patients-year (PY) [7,17,22,26,27,29,32,36]. (**B**) Adverse events incidence rate per 100 PY [7,16,17,22,28,30,33]. (**C**) Serious adverse events incidence rate per 100 PY [9,16,17,20,27]. (**D**) Herpes zoster events incidence rate per 100 PY [9,20,26,30,31,32,33]. Random-effects model was applied. CI, confidence interval.

Colectomy rates were documented in eight studies [7,17,22,26,27,29,32,36] including 838 patients, with an exposure of 622 PY. A total of 49 colectomies were reported (5.8%; 95% CI 4.7–7.0; range 4.4–12.5%). The pooled IR was 13.3 colectomies per 100 PY (95% CI 5.5–21.2; range 4.4–70.1) (Figure 4A). Moderate between-study heterogeneity was observed (I^2^ = 59%).

### 3.9. Upadacitinib Safety

Adverse events (AEs) were evaluated in seven studies reporting 60 AEs in 311 patients (19.3%; 95% CI 14.9–23.7; range 7.1–26.3%) [7,16,17,22,28,30,33], with 112 patient-years (PY) of exposure. The pooled IR was 50.3 AEs per 100 PY (95% CI 18.9–81.7; range 18.6–108.3) (Figure 4B). The heterogeneity between studies was substantial (I^2^ = 70%).

Five studies reported only one serious AE (SAE) in 216 patients (0.5%; 95% CI 0.0–1.4; range 0.0–1.1%) [9,16,17,20,27], with 75 PY of exposure. The pooled IR was 2.3 SAE per 100 PY (95% CI 0.0–5.8; range 0.0–3.9) (Figure 4C). The heterogeneity between studies might not be important (I^2^ = 0%). None of the studies reported major cardiovascular adverse events, and only one patient had a thromboembolic complication classified as SAE.

Seven studies reported a total of three herpes zoster (HZ) infections in 386 patients (0.8%; 95% CI 0.0–1.6; range 0.0–6.3%) [9,20,26,30,31,32,33], with 128 PY of exposure. The pooled IR was 1.7 HZ infections per 100 PY (95% CI 0.0–4.0; range 0.0–19.7) (Figure 4D). Between-study heterogeneity might not be important (I^2^ = 0%).

### 3.10. Sensitivity Analysis

Sensitivity analysis for clinical remission rates in paired studies showed that removing single studies did not significantly alter the pooled effect, confirming the robustness of the results (Table S5).

## 4. Discussion

Data from observational studies on real-world effectiveness and safety provide valuable evidence to support the efficacy observed in RCTs and can help guide therapeutic decision-making in UC. This study is a comprehensive systematic review and meta-analysis of observational studies evaluating the outcomes of upadacitinib in patients with UC, including data from full-text manuscripts and meeting abstracts. The meta-analyses validated the consistent efficacy and safety of upadacitinib for active ulcerative colitis, offering a comprehensive perspective that may assist patients and clinicians in making more informed treatment decisions.

The first observation is the complexity and refractoriness of patients treated with upadacitinib in real-life situations. Our systematic review mostly includes treatment-refractory UC patients: almost 95% of patients were biologic experienced, three out of four had previously failed two or more biologics, more than half had received vedolizumab, and more than half had been exposed to any biologic and tofacitinib. In the RCTs evaluating upadacitinib for UC, nearly half of the patients were anti-TNF naïve, and prior treatment with tofacitinib was not permitted [4]. In the vedolizumab GEMINI 1 [37], ustekinumab UNIFI [38], and tofacitinib OCTAVE induction 1 and 2 [39] RCTs, approximately half of the patients were anti-TNF naïve, and none received a JAKi. In these RCTs, week 8 remission rates with upadacitinib, tofacitinib, ustekinumab, and vedolizumab were consistently lower in anti-TNF experienced patients compared to anti-TNF naïve patients [4,37,38,39]. In real-world settings, a meta-analysis evaluating tofacitinib included 90% of biologic experienced patients, but none had previously received JAKi [40]. In a recent meta-analysis of observational studies with ustekinumab for UC 92% of patients were biologic experienced, and 16% had been exposed to both biologics and tofacitinib [41]. Consequently, this meta-analysis incorporated studies that examined a more challenging population compared to prior RCTs or real-world studies on advanced therapies for UC.

In the short term, upadacitinib induced clinical remission in nearly 70% of patients at week 8, and these remission rates were maintained through weeks 12 to16. The pooled week 8 remission rates reported here were higher than the upadacitinib remission rates observed in the UC induction RCTs [4]. This comparison is not appropriate because the UC1 and UC2 RCTs used a strict definition of remission based on the adapted Mayo total score. In contrast, most of the reviewed observational studies use symptomatic scores such as the simple clinical colitis activity index or the partial Mayo score (Table S4). In addition, clinicians have more flexibility to use concomitant treatments, such as topical therapies, which are prohibited in trials, which may contribute to higher response rates in observational studies. Steroid-free clinical remission rates at week 8 were even higher, although we believe this finding should be treated with caution given the small number of studies evaluating this endpoint. About 80% of patients had a clinical response at week 8 and weeks 12 to 16. Despite the inclusion of patients with complex UC, upadacitinib showed higher short-term clinical remission and response rates compared to real-world effectiveness of vedolizumab at weeks 6 and 14 [42]. Remission rates for upadacitinib at week 8 and weeks 12–16 were also higher than those reported in meta-analyses of the real-world efficacy of tofacitinib and ustekinumab, which employed a similar methodology to our study. Specifically, these studies reported pooled week 8 remission rates of 34.7% for tofacitinib [40] and 45.4% for ustekinumab [41], whereas our study found a remission rate of 68.4% for upadacitinib. Comparisons should be made with caution due to differences in study design and bias in observational studies. These results are consistent with those from a systematic review and network meta-analysis of RCTs evaluating the efficacy and safety of biologics and small molecules for patients with moderate-to-severe ulcerative colitis. In the short term, upadacitinib demonstrated significant superiority over all other interventions and ranked highest for both the induction of clinical remission and endoscopic improvement [43].

One advantage of JAK inhibitors is their rapid onset of action. In particular, upadacitinib provides symptomatic improvement as early as the first day of induction treatment [44]. Our results confirmed the fast onset of action with upadacitinib, with almost half of patients achieving early clinical remission between weeks 2 and 6. Moreover, the effectiveness of upadacitinib is supported by the normalization of objective biomarkers of efficacy. In most studies, mean calprotectin and CRP levels decreased significantly from baseline during induction with upadacitinib. Although endoscopic healing is considered as the most reliable objective measure of drug efficacy in UC, the available endoscopic data in our systematic review were limited and did not allow for meta-analysis. Kaniewska et al. reported that 55.6% of UC patients achieved endoscopic remission at week 8 [18]. In another study, sixteen out of twenty patients (80%) had endoscopic remission, with nine patients (47%) also achieving histological remission after at least 12 weeks of treatment [17].

Due to the recent approval of the drug for UC, there is limited real-world evidence on the long-term outcomes with upadacitinib. This only permits a meta-analysis of clinical remission and response rates at weeks 24 to 36. At this timepoint, 64% of patients included in three studies achieved clinical remission, and 78% of patients in five studies had a clinical response. Despite the small sample size, the mid-term remission and response rates for upadacitinib maintenance were higher than those for vedolizumab, tofacitinib, and ustekinumab in real-world settings [40,41,42]. One limitation of this study is that the majority of the studies included in our systematic review did not offer data on long-term follow-up (i.e., beyond 6 months), despite the significance of sustaining response as a therapeutic objective. However, the rate of clinical remission observed with upadacitinib at weeks 24 to 36 may be indicative of a long-term maintenance of response. Longer-term follow-up in larger real-world cohorts may be useful to further refine the benefit–risk evaluation. In addition, the reviewed studies provide limited data on the need for and outcomes of prolonged induction, dose escalation, and reinduction during maintenance with upadacitinib.

Despite the small study count, subgroup analysis indicated higher short-term effectiveness in biologic-experienced patients. This finding showing the potentiation of upadacitinib in patients with prior exposure to anti-TNF appears to be inconsistent with real-world data on the effectiveness of biologics and tofacitinib, which have consistently shown poorer outcomes in bio-experienced UC patients. Our results are consistent with those of a recent study that reported JAK inhibitors to be more effective in anti-TNF-exposed than in naïve patients, based on six RCTs [45]. Moreover, subgroup analysis evaluated the effectiveness of upadacitinib as a second JAKi for UC. Based on our results, outcomes with upadacitinib were not compromised by prior JAKi failure, with nearly 70% of patients achieving the primary endpoint. Similarly, Farkas et al. recently reported that prior tofacitinib failure did not affect the therapeutic outcome of upadacitinib in UC [46]. Among the studies reviewed, only one indicated that 4% of patients treated with upadacitinib were using concomitant immunosuppressants. It is important to note that the effectiveness of upadacitinib in treating UC was achieved through monotherapy, while in real-world scenarios, many patients receive biologics in combination with immunosuppressants [40,41,42].

Updacitinib showed an acceptable safety profile, consistent with the established safety profile from pivotal RCTs and long-term extension studies in UC, which reported that serious adverse events and adverse events leading to discontinuation of treatment were less frequent in the upadacitinib 45 mg induction group or in the 15 mg or 30 mg maintenance groups than in the placebo group [4,5]. Although selective JAK1 inhibitors such as upadacitinib or filgotinib have been developed to improve the risk-benefit profile of this therapeutic class, safety results from a network meta-analysis of RCTs contradict this notion, as upadacitinib ranked first for all adverse events, and filgotinib ranked third for serious adverse events [43]. However, as a novel finding, the pooled incidence rate (IR) of 2.3 SAE per 100 person-years (PY) observed in our study was lower than the pooled real-world SAE IR previously reported for tofacitinib [40], but slightly higher than that reported for ustekinumab [41]. An increased risk of herpes zoster infections has been observed with all JAK inhibitors. Of note, our study observed a herpes zoster IR of 1.7 per 100 PY, which is significantly lower than the IR of 4.9 PY reported in the open-label extension study for the upadacitinib combined groups [5]. We believe that this reduction may be due to routine vaccination with recombinant zoster vaccine prior to initiation of a JAKi in most studies. In a Canadian observational study of UC patients vaccinated with Shingrix before starting tofacitinib, the IR of herpes zoster was significantly lower than that reported in UC development programs, which appears to be related to the protective effects of zoster vaccination [47]. Although upadacitinib treatment has been associated with increases in lipid levels, none of the studies reported major cardiovascular adverse events and only one patient had a thromboembolic complication classified as SAE. This is consistent with the very low IRs for cardiovascular events and deep venous thrombosis reported in upadacitinib UC development programs [4,5]. The lack of specific safety data in high-risk populations such as the elderly is a limitation of the study.

This meta-analysis has several limitations beyond those inherent in real-world studies. Most studies were retrospective and showed variability in study population, outcome measures, thresholds for clinical remission and response, and the timing of these measurements, making it difficult to compare results across studies meaningfully. Analyses showed significant statistical heterogeneity between studies. A major limitation was the varying definitions of remission and response among studies, similar to other reviews of advanced therapies for UC [40,41,42]. Additionally, there may be publication bias. We were able to calculate Egger’s weighted regression statistic for the primary endpoint, and *p* values indicated no publication bias. The limited number in studies included did not allow us to assess publication bias for several important outcomes. Additionally, the limited duration of follow-up in most studies makes it difficult to obtain reliable data on the long-term outcomes of upadacitinib in UC. To enhance the risk–benefit evaluation of upadacitinib, conducting a meta-analysis of studies with extended follow-up periods and larger real-world cohorts may be essential.

Strengths of the present review process included assessment of the included studies for sources of heterogeneity and risk of bias using gold standard methods. To minimize the risk of publication bias we included studies published as abstracts. The methodology utilized in this meta-analysis included both full articles and meeting abstracts, used the random effects model, and quantified adverse events using IR per 100 PY as a function of time elapsed. This approach is consistent with two previous meta-analyses conducted by our group on other advanced therapies for patients with UC. With appropriate considerations, this has allowed indirect comparisons of the real-world effectiveness and safety of recently approved drugs for UC.

## 5. Conclusions

In conclusion, the results of this meta-analysis of observational studies confirm the effectiveness and safety of upadacitinib in a highly treatment-refractory population of patients with active moderate-to-severe UC. These findings reinforce upadacitinib’s favorable long-term benefit–risk profile in UC treatment. The real-world outcomes of upadacitinib in UC are consistent with clinical trials and provide information to guide therapeutic decisions in clinical practice.

## Figures and Tables

**Table 1:** Demographics of populations, characteristics of ulcerative colitis (UC) and treatments. SD, standard deviation; LFU, last follow-up; BMI, body mass index; Extension: E1, proctitis, E2, left-sided, E3, extensive; Bio, biologic; TNF, tumor necrosis factor; VED, vedolizumab; UST, ustekinumab; TOF, tofacitinib.

Study	No. Patients	Mean LFU (Weeks)	Sex Female (%)	Mean Age	Smokers (%)	Mean BMI (kg/m^2^)	Mean UC Duration (Years)	Extension Montreal Classification (%)			Prior Treatment (%)							Current Steroids
								E1	E2	E3	Total Bio	=1 Bio	≥2 Bio	Anti-TNF	VED	UST	TOF	
Dalal et al. [7]	76	33.3	47.4	39.3	1.3	25.0	9.0	-	-	-	100	-	-	90.8	73.7	30.3	30.3	53.9
Friedberg et al. [8]	44	6.9	47.7	38.9	4.5	-	12.4	7.5	32.5	60.0	100	18.2	81.8	100	61.4	36.4	38.6	31.8
Cleveland et al. [9]	57	31.7	43.9	40.7	-	-	11.0	7.0	35.1	57.9	98.2	8.8	89.5	-	-	-	42.1	-
Yin et al. [16]	8	35.0	37.5	34.5	-	-	9.1	0.0	25.0	75.0	100	12.5	87.5	-	50.0	25.0	-	12.5
Choon et al. [17]	42	5.3	47.6	39.0	-	-	-	6.7	73.8	19.0	92.9	40.5	52.4	-	-	-	-	-
Kaniewska et al. [18]	27	8.0	-	-	-	-	-	-	-	-	-	-	-	-	-	-	-	-
Zeissig et al. [19]	124	8.0	39.5	38.6	4.8	23.9	7.0	5.6	46.0	48.4	85.5	20.2	52.4	-	-	-	-	46.0
Al-Zarrad et al. [20]	22	12.0	27.3	-	-	-	-	-	-	-	-	-	-	-	-	-	-	-
Bhatia et al. [21]	34	-	47.1	37.4	-	-	12.0	-	-	-	100	29.4	70.6	-	-	-	-	-
Teani et al. [22]	12	13.1	-	-	-	-	-	-	-	-	100	16.7	83.3	100	-	-	25.0	25.0
Garcia et al. [23]	32	-	-	-	-	-	-	-	-	-	100	-	-	-	-	-	78.1	-
Harris et al. [24]	34	8.0	-	-	-	-	-	-	-	-	67.6	-	-	-	-	-	-	-
Annadurai et al. [25]	11	-	-	-	-	-	-	0.0	18.2	81.8	-	-	-	-	-	-	-	-
Patel et al. [26]	98	11.5	35.7	26.3	-	-	6.0	-	-	68.4	69.4	13.3	56.1	-	-	-	31.6	-
Chowla et al. [27]	87	15.4	37.9	40.5	-	-	-	-	-	-	100	-	-		-	-	19.5	-
Doumas et al. [28]	15	10.8	46.7	33.9	-	24.9	7.8	13.3	0.0	86.7	100	-	-	-	-	-	-	-
Kochhar et al. [29]	526	52.0	44.9	40.4	4.8	-	-	3.4	16.2	80.4	-	-	-	34.4	15.4	21.5	-	27.9
Gilmore et al. [30]	152	16.0	-	-	-	-	-	-	-	-	-	-	-	-	-	-	27.6	-
Boneschansker et al. [31]	35	12.0	54.3	39.0	-	-	9.6	11.4	28.6	60.0	-	-	-	97.1	74.3	40.0	34.3	40.0
Levine et al. [32]	16	34.9	43.8	37.5	-	-	7.9	0.0	25.0	75.0	100	0.0	100	-	43.8	25.0	100	50.0
Hosomi et al. [33]	6	8.0	33.3	45.2	-	-	6.4	0.0	0.0	100	100	16.7	83.3	50.0	33.3	33.3	50.0	-
Radia et al. [34]	5	4.0	0.0	-	-	-	6.0	-	-	-	100	-	-	-	-	-	100	100
Cleveland et al. [35]	18	4.4	38.9	39.3	5.6	-	15.2	6.3	37.5	56.3	100	0.0	100	-	-	-	100	44.4
Odah et al. [36]	26	52.8	53.8	35.2	0.0	-	-	3.8	15.4	80.8	100	-	-	-	76.9	69.2	100	57.7
Total of patients	1388																	
Mean		19.6	40.0	37.7	3.1	24.6	8.3	4.7	25.8	69.5	94.6	17.6	75.0	78.7	53.6	35.1	53.2	44.5

## Data Availability

No new data were created.

## Supplementary Materials

The following supporting information can be downloaded at: https://www.mdpi.com/article/10.3390/jcm14072232/s1, Table S1: Search queries used in PubMed; Table S2: Characteristics of studies; Table S3: Critical appraisal of studies according to the Joanna Briggs Institute for prevalence and incidence studies criteria; Table S4: Definitions of clinical remission, steroid-free clinical remission, clinical response and biochemical remission; Table S5: Sensitivity analyses showing the influence of each study on the pooled rates of clinical remission; Figure S1: Clinical response rate at weeks 2–6, 8, and 12–16; Figure S2: Steroid-free clinical remission rate at week 8; Figure S3: Clinical remission rate at weeks 8 and 12–16 in patients with prior Janus kinase inhibitor treatment; Figure S4: Subgroup analysis for clinical remission rate at week 8.
