# Supplementary material for: Real-World Effectiveness and Safety of Upadacitinib in Patients with Ulcerative Colitis: A Systematic Review and Meta-Analysis
Source: J Clin Med. 2025 Mar 25;14(7):2232. doi: 10.3390/jcm14072232 (PMC11989280; doi:10.3390/jcm14072232)

Journal of Clinical Medicine

MDPI

Taxonera C, et al. Real-world effectiveness and safety of upadacitinib in patients with ulcerative colitis: a systematic review and meta-analysis.

Supplementary material for peer review

Supplementary Table S1: Search queries used in PubMed

The following exact boolean search was used in Medline (PubMed):

(ulcerative colitis[Title/Abstract]) AND ((upadacitinib[Title/Abstract]) OR (janus kinase inhibitor[Title/Abstract]) OR (JAK inhibitor[Title/Abstract]) OR (JAK-STAT[Title/Abstract]) OR (Rinvoq[Title/Abstract]))

No filter for study design, language or year of publication was used.

Supplementary Table S2. Characteristics of studies. Effectiveness outcomes assessed at week 2-6, week 8, week 12-16, week 24-36 and last follow-up (LFU). Effectiveness outcomes: 1) Clinical remission, 2) Clinical response, 3) Steroid-free clinical remission, 4) Treatment failure, 5) Biochemical remission. BR, brief Report; Art, article; Abs, abstract; CR, case Report; P, prospective; R, retrospective; CS, case series; C, cohort; SC, single center; MC, multi-center; AEs, adverse events; SAEs, severe adverse events.

Study	Year	Country	Type	Study Design			N° patients	Effectiveness Outcomes (time)					Safety Outcomes			
				P/R	CS/C	SC/MC		Weeks 2-6	Week 8	Weeks 12-16	Weeks 24-36	LFU	AEs	SAEs	H. zoster	Colectomy
Dalal et al. [7]	2023	USA	BR	R	C	MC	76		5	1, 2		4	Yes	No	No	Yes
Friedberg et al. [8]	2023	USA	Art	P	C	SC	44	1, 2	1, 2, 3, 5			4	No	No	No	No
Cleveland et al. [9]	2024	USA	Abs	P	C	SC	57				1		No	Yes	Yes	No
Yin et al. [16]	2024	USA	CR	R	CS	SC	8		5		1	4	Yes	Yes	No	No
Choon et al. [17]	2024	UK	Abs	R	C	SC	42	1, 2	5			4	Yes	Yes	No	Yes
Kaniewska et al. [18]	2024	Poland	Abs	P	C	MC	27	1	1				No	No	No	No
Zeissig et al. [19]	2024	Europe	Abs	P	C	MC	124	1	1, 3, 5			4	No	No	No	No
Al-Zarrad et al. [20]	2024	UK	Abs	P	C	SC	22		1, 2, 5			4	No	Yes	Yes	No
Bhatia et al. [21]	2024	USA	Abs	R	C	SC	34						No	No	No	No
Teani et al. [22]	2024	Italy	Abs	R	C	MC	12		1, 2			4	Yes	No	No	Yes
Garcia et al. [23]	2024	Spain	Abs	R	C	MC	32			1			No	No	No	No
Harris et al. [24]	2024	UK	Abs	P	C	MC	34		1, 2			4	No	No	No	No
Annadurai et al. [25]	2024	USA	Abs	R	C	SC	11						No	No	No	No
Patel et al. [26]	2023	USA	Abs	R	C	MC	98		1, 2	1, 2			No	No	Yes	Yes
Chowla et al. [27]	2023	USA	Abs	R	C	MC	87			2		4	No	Yes	No	Yes
Doumas et al. [28]	2023	USA	Abs	R	C	MC	15			2		4	Yes	No	No	No
Kochhar et al. [29]	2024	USA	Art	R	C	MC	526						No	No	No	Yes
Gilmore et al. [30]	2024	Australia	Abs	R	C	MC	152		1	1		4	Yes	No	Yes	No
Boneschansker et al. [31]	2023	USA	Art	R	C	SC	35			1, 2		4	No	No	Yes	No
Levine et al. [32]	2023	USA	BR	R	CS	SC	16				1	4	No	No	Yes	Yes
Hosomi et al. [33]	2023	Japan	CR	R	CS	SC	6		1			4	Yes	No	Yes	No
Radia et al. [34]	2023	UK	Abs	R	CS	SC	5	1, 2				4	No	No	No	No
Cleveland et al. [35]	2023	USA	Abs	P	C	SC	18		1	1			No	No	No	No
Odah et al. [36]	2024	USA	Art	R	C	MC	26		2, 3				No	No	No	Yes
**Total of patients**							**1388**									

Supplementary Table S3. Critical appraisal of included studies according to the Joanna Briggs Institute (JBI) for prevalence and incidence studies criteria. The risk of bias of the studies was categorized as low risk of bias (70% or more "yes" responses), moderate risk of bias (50%–69% "yes" responses) and high risk of bias (up to 49% "yes" responses). N/A, not applicable.

Study	Was the sample representative of the target population?	Were study participants recruited in an appropriate way?	Was the sample size adequate?	Were the study subjects and the setting described in detail?	Was the data analysis conducted with sufficient coverage of the identified sample?	Were objective, standard criteria used for the measurement of the condition?	Was the condition measured reliably?	Was there appropriate statistical analysis?	Are all important confounding factors/subgroups /differences identified and accounted for?	Were subpopulations identified using objective criteria?	Total	Risk of bias
Dalal et al. [7]	Yes	Yes	Yes	Yes	Yes	Yes	Yes	Yes	Yes	Yes	100%	LOW
Friedberg et al. [8]	Yes	Yes	Yes	Yes	Yes	Yes	Yes	Yes	Yes	Yes	100%	LOW
Cleveland et al. [9]	Yes	Yes	Yes	Yes	Yes	Yes	Yes	Yes	Unclear	Yes	90%	LOW
Yin et al. [16]	Yes	Yes	No	Yes	Yes	Yes	Yes	Yes	Yes	Yes	90%	LOW
Choon et al. [17]	Yes	Yes	Yes	Yes	Yes	Yes	Yes	Unclear	Yes	Yes	90%	LOW
Kaniewska et al. [18]	Yes	Yes	Yes	No	Yes	Yes	Unclear	Unclear	No	No	50%	MODERATE
Zeissig et al. [19]	Yes	Yes	Yes	Yes	Yes	Yes	Yes	Unclear	Unclear	Yes	70%	LOW
Al-Zarrad et al. [20]	No	Yes	Yes	No	Yes	Yes	Yes	Unclear	No	No	50%	MODERATE
Bhatia et al. [21]	Yes	Yes	Yes	Yes	Yes	Yes	Unclear	Yes	Unclear	Yes	80%	LOW
Teani et al. [22]	Unclear	Yes	Yes	Yes	Yes	Yes	Unclear	Yes	Yes	Yes	80%	LOW
Garcia et al. [23]	Unclear	Yes	Yes	No	Yes	Yes	Unclear	Yes	No	Unclear	50%	MODERATE
Harris et al. [24]	Unclear	Yes	Yes	No	Yes	Yes	Unclear	Yes	No	Unclear	50%	MODERATE
Annadurai et al. [25]	Unclear	Yes	Yes	No	Yes	Yes	N/A	N/A	No	Yes	50%	MODERATE
Patel et al. [26]	Yes	Yes	Yes	Yes	Yes	Yes	Yes	Yes	Yes	Yes	100%	LOW
Chowla et al. [27]	Yes	Yes	Yes	No	Yes	Yes	Yes	Unclear	Unclear	Unclear	60%	MODERATE
Doumas et al. [28]	Yes	Yes	Yes	Yes	Yes	Yes	Unclear	Unclear	No	Yes	70%	LOW
Kochhar et al. [29]	Yes	Yes	Yes	Yes	Yes	Yes	Unclear	Yes	Yes	Yes	90%	LOW
Gilmore et al. [30]	Unclear	Yes	Yes	No	Yes	Yes	Yes	Unclear	Yes	Unclear	60%	MODERATE
Boneschansker et al. [31]	Yes	Yes	Yes	Yes	Yes	Yes	Yes	Yes	Yes	Yes	100%	LOW
Levine et al. [32]	Yes	Yes	Yes	Yes	Yes	Yes	Yes	Unclear	Yes	Yes	90%	LOW
Hosomi et al. [33]	Yes	Yes	No	Yes	Yes	Yes	Yes	Unclear	Yes	Yes	80%	LOW
Radia et al. [34]	No	Yes	No	Yes	Yes	Yes	Yes	Unclear	Yes	Yes	70%	LOW
Cleveland et al. [35]	Yes	Yes	Yes	Yes	Yes	Yes	Unclear	Unclear	Yes	Yes	80%	LOW
Odah et al. [36]	Yes	Yes	Yes	Yes	Yes	Yes	Yes	Yes	Yes	Yes	100%	LOW

Supplementary Table S4. Definitions of clinical remission, steroid-free clinical remission (SFCR), clinical response and biochemical remission. Definitions of clinical remission, SFCR, clinical response and biochemical remission were classified into 3 categories, from 0 = least stringent to 2 = most stringent. SCCAI, simple clinical colitis activity index; PMS, partial Mayo score; CRP, C-reactive protein; FCP, fecal calprotectin; SFS, stool frequency sub-score; RBS, rectal bleeding sub-score. *Studies by Zeissig et al [19] and Al-Zarrad et al [20] reported combined FCP and CRP biochemical remission.

Author	Clinical remission	Strictness	SFCR	Strictness	Clinical response	Strictness	Biochemical remission	Strictness
Dalal et al. [7]	Steroid-free + SCCAI ≤ 2 or PMS ≤ 2 or Documentation	2	Steroid-free + SCCAI ≤ 2 or PMS ≤ 2 or Documentation	2	Reduction SCCAI or Mayo score ≥ 3 or Documentation	2	CRP < 10 mg/L	1
Friedberg et al. [8]	SCCAI < 3	2	Systemic steroid-free + Reduction SCCAI ≥ 3	2	Reduction SCCAI ≥ 3	2	FCP < 250 μg/g<br>CRP < 5 mg/L	2
Cleveland et al. [9]	SCCAI < 3	2			Reduction SCCAI ≥ 3	2		
Yin et al. [16]	Steroid-free + PMS < 2	2	Steroid-free + PMS < 2	2			FCP < 250 μg/g<br>CRP < 5 mg/L	2
Choon et al. [17]	SCCAI ≤ 2	2			Reduction SCCAI ≥ 3	2	FCP < 150 μg/g	1
Kaniewska et al. [18]	Not defined	NA						
Zeissig et al. [19]*	SFS ≤ 1 + RBS = 0	1	Steroid-free + SFS = ≤ 1 + RBS = 0	1	Reduction PMS ≥ 2.5	2	FCP ≤ 250 μg/g<br>**and** CRP < 5 mg/L	2
Al-Zarrad et al. [20]*	SCCAI < 2.5	2			Reduction SCCAI ≥ 3	2	FCP < 250 μg/g<br>**and** CRP < 5 mg/L	2
Bhatia et al. [21]						NA		
Teani et al. [22]	Not defined	NA			Not defined			
Garcia et al. [23]	Not defined	NA						
Harris et al. [24]	Not defined	NA			Not defined	NA		
Annadurai et al. [25]								
Patel et al. [26]	No symptoms	0			Symptoms reduction > 50%	1		
Chowla et al. [27]					Symptoms reduction + Good general well being	0		
Doumas et al. [28]					Not defined	NA		
Kochhar et al. [29]								
Gilmore et al. [30]	SFS ≤ 1 + RBS = 0	1						
Boneschansker et al. [31]	Steroid-free + SCCAI ≤ 2 or No bleeding and diarrhea	2	Steroid-free + SCCAI ≤ 2 or No bleeding and diarrhea	2	Symptoms reduction but no remission criteria	0		
Levine et al. [32]	PMS ≤ 2	2	≥ 30 days steroid-free + PMS ≤ 2	2	Reduction PMS ≥ 1 + Reduction RBS ≥ 1 or RBS ≤ 1	2		
Hosomi et al. [33]	PMS < 2	2						
Radia et al. [34]	SCCAI < 3	2			Reduction SCCAI ≥ 3	2		
Cleveland et al. [35]	SCCAI < 3	2						
Odah et al. [36]			Steroid-free + Gastrointestinal symptoms reduction	0	Gastrointestinal symptoms reduction	0		

Supplementary Table S5. Sensitivity analyses showing the influence of each study on the pooled rates of clinical remission at weeks 2 to 6 (A), week 8 (B), weeks 12 to 16 (C) and weeks 24 to 36 (D). When single studies were omitted one by one, the pooled effect did not change significantly, further supporting the robustness of the results.

(A) Weeks 2-6 clinical remission

Study omitted	Estimate	[95% Conf. Interval]
Friedberg 2023	0.4307	[0.3141; 0.5507]
Choon 2024	0.4510	[0.2886; 0.6182]
Kaniewska 2024	0.5252	[0.3566; 0.6912]
Zeissig 2024	0.4975	[0.2881; 0.7074]
Radia 2023	0.5068	[0.3622; 0.6508]
**Pooled**	**0.4834**	**[0.3424; 0.6257]**

(B) Week 8 clinical remission

Study omitted	Estimate	[95% Conf. Interval]
Friedberg 2023	0.6670	[0.5264; 0.7950]
Kaniewska 2024	0.7050	[0.5657; 0.8285]
Zeissig 2024	0.6941	[0.5319; 0.8366]
Al-Zarrad 2024	0.6676	[0.5278; 0.7950]
Teani 2024	0.6697	[0.5324; 0.7947]
Harris 2024	0.6907	[0.5456; 0.8202]
Patel 2023	0.7266	[0.6455; 0.8015]
Gilmore 2024	0.6661	[0.5265; 0.7934]
Hosomi 2023	0.6737	[0.5397; 0.7955]
**Pooled**	**0.6844**	**[0.5550; 0.8018]**

(C) Weeks 12-16 clinical remission

Study omitted	Estimate	[95% Conf. Interval]
Dalal 2023	0.7274	[0.5318; 0.8872]
Garcia 2024	0.6927	[0.4984; 0.8577]
Patel 2023	0.6832	[0.4813; 0.8556]
Gilmore 2024	0.6655	[0.4923; 0.8193]
Boneschansker 2023	0.7754	[0.6648; 0.8698]
**Pooled**	**0.7105**	**[0.5523; 0.8470]**

(D) Weeks 24-36 clinical remission

Study omitted	Estimate	[95% Conf. Interval]
Cleveland 2024	0.6143	[0.1174; 0.9960]
Yin 2024	0.5523	[0.2187; 0.8633]
Levine 2023	0.7463	[0.5950; 0.8752]
**Pooled**	0.6460	[0.3671; 0.8844]

Supplementary Figure S1 (A). Clinical response rate at weeks 2-6. Random-effects model was applied. ES, effect size; CI, confidence interval.

Weeks 2-6 Clinical response rate (%)

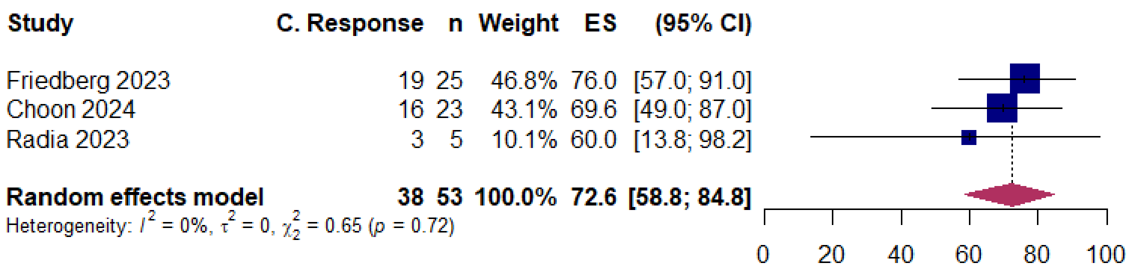

Supplementary Figure 1 (B). Clinical response rate at week 8.

Week 8 Clinical response rate (%)

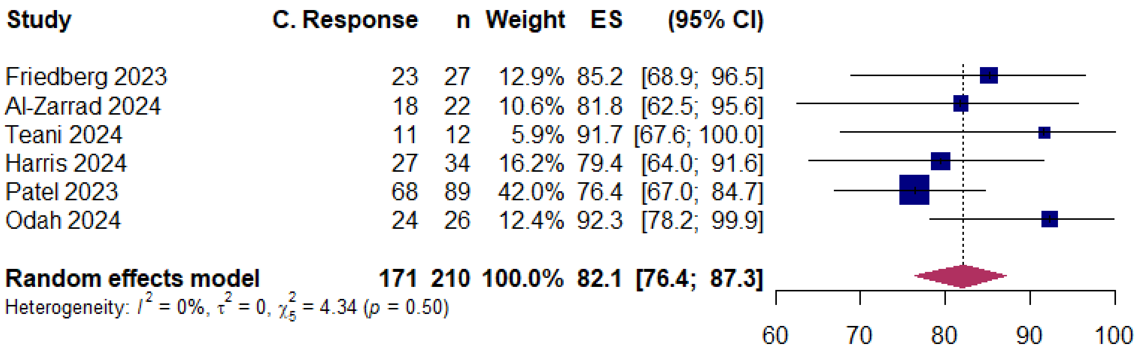

Supplementary Figure S1 (C). Clinical response rate at weeks 12-16.

Weeks 12-16 Clinical response rate (%)

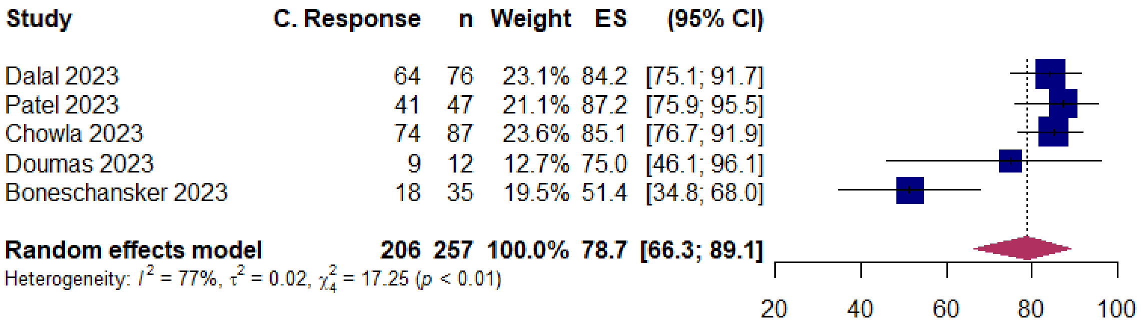

Supplementary Figure S2. Steroid-free clinical remission (SFCR) rate at week 8. Random-effects model was applied. ES, effect size; CI, confidence interval.

Week 8 SFCR rate (%)

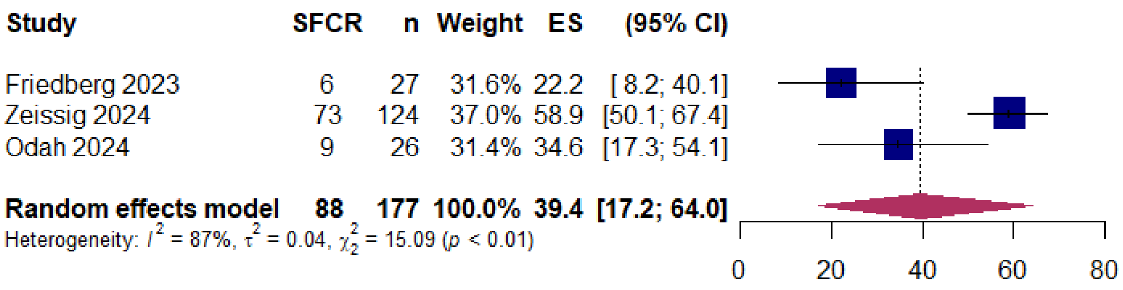

Supplementary Figure S3 (A). Clinical remission rate at week 8 in patients with prior Janus kinase inhibitor (JAKi) treatment. Random-effects model was applied. ES, effect size; CI, confidence interval.

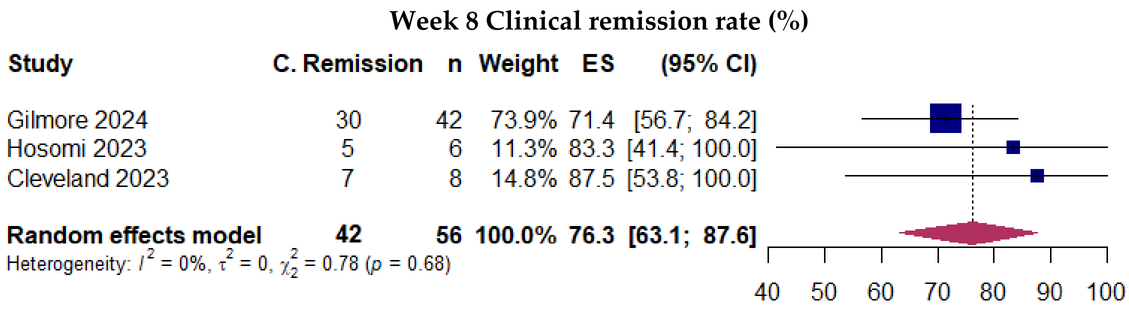

Supplementary Figure S3 (B). Clinical remission rate at weeks 12-16 in patients with prior Janus kinase inhibitor (JAKi) treatment. Random-effects model was applied. ES, effect size; CI, confidence interval.

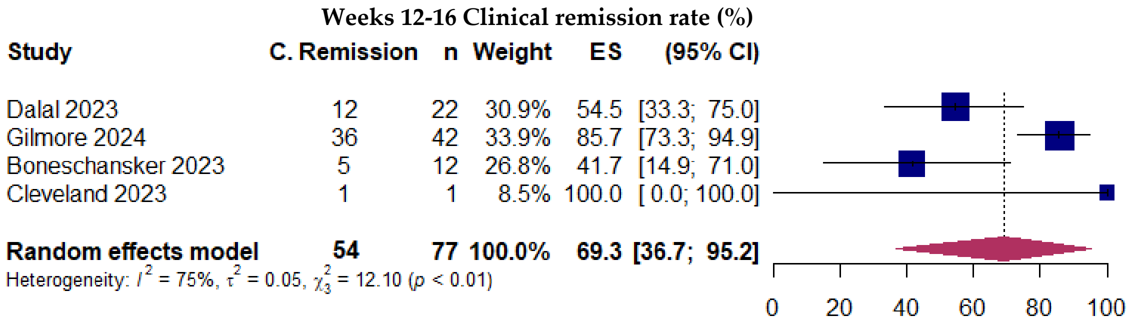

Supplementary Figure S4. Subgroup analysis for clinical remission rate at week 8. Random-effects model was applied. ES, effect size; CI, confidence interval.

A. Study design: prospective vs retrospective.

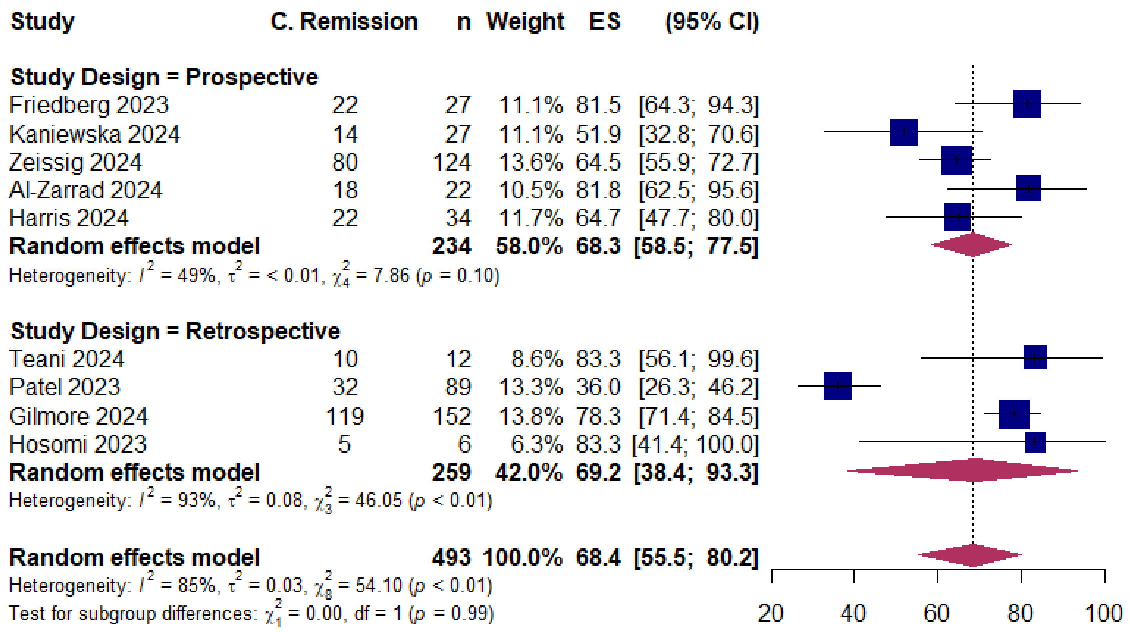

B. Location: single center vs multi-center.

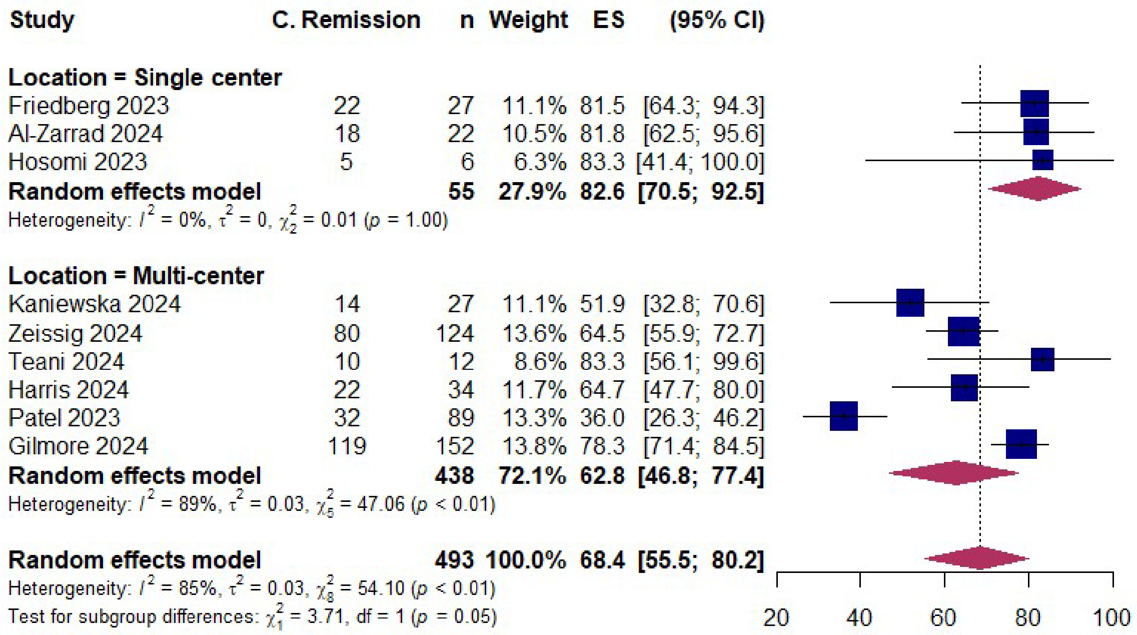

C. Percentage of patients with prior exposure to biologics: 100% vs. ≤ 85%

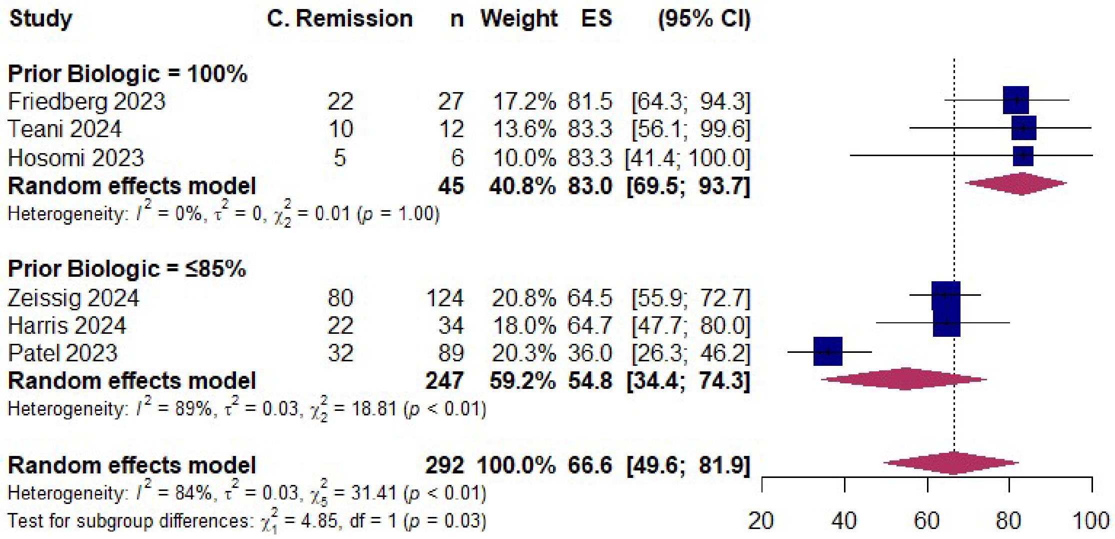

D. 100% prior exposure to JAKi: No vs Yes.

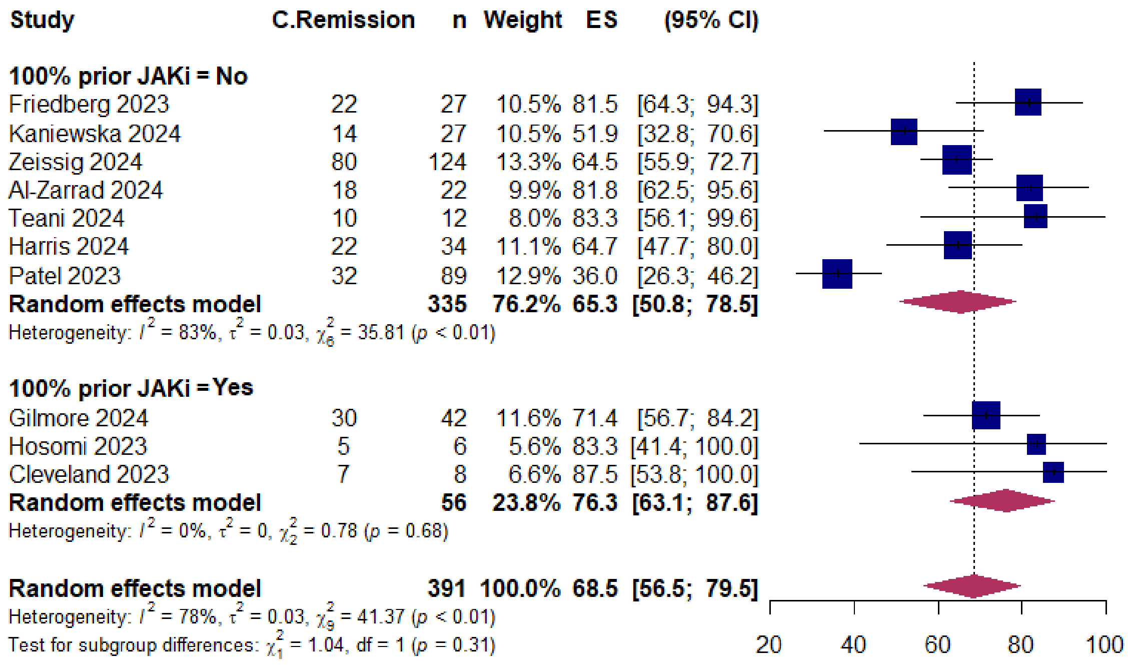

Supplement: Supplementary file 1 [file jcm-14-02232-s001.zip › jcm-3519463-supplementary.pdf]
